# Association study of two single nucleotide polymorphisms rs10757278 and rs1333049 with atherosclerosis, a case-control study from Iraq

**DOI:** 10.22099/mbrc.2019.33818.1406

**Published:** 2019-09

**Authors:** Ahmed Abduljabbar Suleiman, HananYassin Muhsin, Rafid A. Abdulkareem, Farah Amer Abed

**Affiliations:** 1 University of Anbar, College of Science, Iraq; 2 University of Baghdad, College of Education for Pure Sciences (Ibn- AL-Haitham); 3 Department of Genetic Engineering, Institute of Genetic Engineering and Biotechnology for Post Graduate Studies, University of Baghdad, Baghdad, Iraq; 4 Ministry of Science and Technology, Baghdad, Iraq

**Keywords:** rs10757278, rs1333049, Tetra ARMS, CAD, SNPs

## Abstract

Atherosclerosis is one of the most important coronary artery disease (CAD) caused by lipid accumulation, hypertension, smoking, and many other factors such as environmental and genetic factors. It has been recorded that genetic variations in rs10757278 and rs1333049 are correlated with CAD. In the present study, 100 blood samples were collected (50 CAD patients and 50 appeared to be healthy controls), who referred to Ibn-Albytar general hospital/in Bagdad city for heart disease from February to March 2019. Genotyping for two SNPs rs10757278 and rs1333049, were done by Tetra ARMS method. For the rs10757278 polymorphism, the GG genotype verses to AA genotype was significantly associated with the risk of CAD (OR=5.16, 95% CI:1.02-26.0, P=0.047). For the rs10757278 polymorphism, there was no significant associatin between genotypes and the susceptibiulity to CAD. In the present study, the rs10757278 polymorphism showed an association with CAD.

## INTRODUCTION

Atherosclerosis is a reformer ailment of the blood vessels as an development of heart disease, an incipient incision in the artery endothelial induced by environmental and genetic factors [1]. Cardiovascular diseases are one of the most important causes of death in the developing countries and many genetic alterations are renowned to manipulate coronary artery disease (CAD) [2].

Several studies found a strong correlation between many single nucleotide polymorphisms and many heart diseases [3]. Abundant of genome wide association studies have exposed that rs10757278 polymorphism is connected to heart diseases like atherosclerosis, myocardial infarction. This polymorphism is located on chromosome 9p21 near the tumor suppressor gene *CDKN2A* and *CDKN2B*. Many studies on Caucasians, Asian, and European ancestry, reported that there is a significant association of rs10757278 with heart diseases [4, 5].

Globally, there are numerous polymorphisms on chromosome 9p21 seqment. However, only rs1333049 is correlated with CAD, which was first conveyed by Samani et al. (2007) [6]. It was first reported in the German people that the C allele of this polymorphism is substituted for G nucleotide. This work aimed to evaluate the role of these polymorphisms in Iraqi patients with atherosclerosis in a case-control study.

## MATERIALS AND METHODS

Peripheral  blood specimens were collected from 100 subjects, 50 CAD patients who referred to Ibn-Albytar general hospital for heart disease from February to March 2019 and 50 appeared to be healthy controls with no family history of heart disease. Patients were chosen according to the coronary angiography and electrocardiogram (ECG) criteria. DNA was extracted from blood samples using a mammalian genomic DNA extraction kit (Geneaid Biotech). It was checked for integrity by agarose gel electrophoresis, and the purity and concentration were checked by nanodrop. The extracted DNA stored at -20°C until use. For Tetra ARMS technique the online tool was used http://primer1.soton.ac.uk/primer1.html. Primers were listed in Table 1. The PCR was performedusing BIONEER primex PCR ready to use tube and the condition can be seen in Table 1.

**Table 1:** Primer sequence for SNPs detection by TETRA ARMS

**Sequence 5—3**	**Product **	**Annealing Temperature**
IF AGGGTGTGGTCATTCCGGGAG	For G 257bp for A 325bp two outer 532bp	61°C
IR CTACTCTGTCTTGATTCTGCATCGCTTCT		
OF CTGAGGTCGCAACTAAAAGCCAAGATT		
OR CGCTGTTCCCAAGTAGCCAGGATA		
IF CCTCATACTAACCATATGATCAACAGATC	For C 234bp For G 296bp two outer 481bp	60°C
IR TCTGCGAGTGGCTGCTTATC		
OF AAGTAAAAAAAGAATGGGCTGCTG		
OR TGAGCATAGCTGTAAAACAAAGGG		

The results of PCR reactions were run on gel and were collected for the statistical analysisThe results were checked for the Hardy-Weinberg equilibrium. Alleles and genotypes of rs10757278 and rs1333049 polymorphisms were given as percentage frequencies and significant differences between patients and controls were assessed by the the odds ratio (OR) and its 95% CI (confidence interval). The WinPepi software version 11.65 was used to obtain these estimations.

## RESULTS AND DISCUSSION

Table 2 shows the genotypic and alleleic distributions in CAD and control groups. The observed genotypic frequency of the study polymorphisms in the control subjects did not show significant deviation from the expected values based on the Hardy-Weinberg equilibrium (For rs10757278: χ^2^=2.93, df=1, P=0.086; For rs1333049: χ^2^=0.23, df=1, P=0.594). 

The result also showed that the rs10757278 was more distributed in Iraqi patients with atherosclerosis. For the rs10757278 polymorphism, the AG genotype did not showed significant association with the risk of CAD (OR=1.77, 95% CI: 0.63-4.94, P=0.273). The GG genotype verses to AA genotype was significantly associated with the risk of CAD (OR=5.16, 95% CI:1.02-26.0, P=0.047) (Table 2). It should be noted that there was a significant linear trend between the number of the G allele and the risk of CAD (χ^2^=5.08, P=0.024). For the rs10757278 polymorphism, there was no significant associatin between genotypes and the susceptibiulity to CAD (Table 2). 

**Table 2:** Genotypic distribution of the rs10757278 and rs1333049 polymorphisms among CAD cases and healthy controls

Polymorphisms	Controls	Cases	OR	95% CI	P-value
rs10757278	-	-	-	-	-
AA	40	31	1.0	-	-
AG	8	11	1.77	0.63-4.94	0.276
GG	2	8	5.16	1.02-26.0	0.047
rs1333049					
GG	23	19	1.0	-	-
GC	23	22	1.15	0.49-2.69	0.723
CC	4	9	2.72	0.72-10.2	0.138

In the present study, the rs10757278 showed an association with CAD. These results were consistent with those of suggested that rs1333040, rs1333049, rs2383206, rs2383207, rs10757274 and rs10757278 variants may be associated with susceptibility to CAD [7]. Same findings were reported that s1333040, rs1333049, rs2383206, rs2383207, rs10757274, and rs10757278 polymorphisms may serve as genetic biomarkers of CAD in East Asians [8]. Moreover, rs2383206, rs10757274, and rs10757278 polymorphisms may also serve as genetic biomarkers of CAD in Caucasians and west Asians.
